# Editorial: Cancer prevention, treatment and survivorship in the LGBTQIA community

**DOI:** 10.3389/fonc.2023.1227911

**Published:** 2023-07-11

**Authors:** Jane M. Ussher, Gwendolyn P. Quinn, Janette Perz

**Affiliations:** ^1^ Translational Health Research Institute, School of Medicine, Western Sydney University, Sydney, NSW, Australia; ^2^ Department of Obstetrics and Gynecology and Population Health, New York University (NYU) Grossman School of Medicine, New York, NY, United States

**Keywords:** cancer, minority stress, LGBTQI, health care professional, adolescent and young adult (AYA) cancer, trans, intersex (LGBQTI), social support

## 1 Introduction

Sexuality and gender minorities (SGM), including those who identify as lesbian, gay, bisexual, transgender, queer, and intersex (LGBTQI+), constitute a growing and underserved population in the realm of cancer care. This community faces a greater burden of cancer (1–3) and encounters distinctive psychosocial challenges. These challenges include elevated rates of cancer-related distress and sexual concerns (2, 4, 5), reduced quality of life (QOL) (6), and diminished support from their biological families (7), when compared to non-LGBTQI+ individuals with cancer and their caregivers. Concurrently, LGBTQI+ individuals also experience heightened dissatisfaction with cancer healthcare (8, 9), which encompasses difficulties in communication with healthcare professionals (HCPs) (10), barriers in accessing cancer services (8), and a lack of LGBTQI+-inclusive cancer information or support (2, 11). Revealing their sexual orientation or gender identity (SOGI) to HCPs is a significant source of distress due to concerns about potential hostility or cis-heteronormative biases that might result in substandard care (2, 10, 12, 13). However, if SOGI is not disclosed, LGBTQI+ individuals with cancer are more likely to report unmet needs, a sense of invisibility, dissatisfaction with care, and poor psychological well-being (10, 14, 15).

The American Society of Clinical Oncology has acknowledged the existence of this healthcare disparity and determined that there is inadequate understanding of the healthcare requirements, outcomes, lived experiences, and effective interventions to enhance outcomes for LGBTQI+ communities (1). Consequently, healthcare providers and policymakers lack the necessary tools to establish inclusive and culturally-sensitive programs aimed at prevention, guidance, and support for LGBTQI+ individuals with cancer and their families (16, 17).

This Research Topic of Frontiers in Oncology aims to bridge the “knowledge-to-action” gap by bringing together cutting-edge research that explores the experiences of cancer survivorship and cancer care within the LGBTQI+ population. The Research Topic encompasses original studies utilizing quantitative, qualitative, and mixed methods designs. While previous research on LGBTQI+ cancer has primarily focused on cisgender lesbian women and gay men with breast or prostate cancer or, we expand upon this by including research on underrepresented communities. This includes LGBTQI+ individuals with various tumor types, transgender individuals (both binary and non-binary), LGBTQI+ adolescents and young adults (AYAs), LGBTQI+ individuals from diverse racial and cultural backgrounds, individuals with an intersex variation, and LGBTQI+ informal cancer caregivers. Through this research, we aim to address significant gaps in the existing literature, representing a pioneering effort to identify the concerns and experiences of this previously marginalized population of cancer survivors and their informal caregivers.

In this special edition, we also feature recent research that explores the perspectives of healthcare practitioners (HCPs) who work with LGBTQI+ individuals with cancer. Examining the beliefs and knowledge of oncology HCPs is crucial for identifying barriers and facilitators to culturally safe and inclusive LGBTQI+ cancer care (16). HCPs who possess greater knowledge of LGBTQI+ healthcare needs exhibit more positive attitudes, intentions, and behaviors toward LGBTQI+ cancer patients (18). They recognize the importance of acquiring patients’ sexual orientation and gender identity (SOGI) information, avoiding cis-heteronormative assumptions by not assuming all patients are heterosexual and cisgender (i.e., identifying with the gender assigned at birth), and being willing to be recognized as LGBTQI+friendly providers (19–21). However, surveys conducted among oncology radiation therapists (22), physicians (20, 23, 24), nurses, and other advanced care professionals (19, 21) consistently reveal low levels of knowledge about LGBTQI+ patients. Consequently, training programs have been developed for HCPs to enhance cultural safety for LGBTQI+ individuals with cancer, with the goal of fostering inclusive and affirming cancer care (25, 26).

The research findings presented in this special edition will contribute to a better understanding of this often-overlooked population in cancer care. They will inform the development of future training programs, as well as provide policy and practice recommendations. A summary of the papers featured in this special edition is provided below. In describing the papers, we utilize the language employed by the authors to depict their study samples - SGM or LGBTQI+.

## 2 Summary of papers

This special edition of Frontiers includes several papers that investigate the levels of distress and quality of life among LGBTQI+ individuals with cancer, shedding light on the factors associated with these outcomes. Ussher et al. examined the psychosocial factors linked to distress and quality of life among LGBTQI+ individuals with cancer, drawing upon the quantitative findings of the Out with Cancer Study. The research reveals that 41% of LGBTQI+ individuals with cancer reported high or very high levels of distress, which is three to six times higher compared to previous studies conducted among non-LGBTQI individuals with cancer. The study also identifies higher rates of distress among LGBTQI+ individuals who are AYAs, transgender, bisexual, queer, and those residing in rural areas. The elevated distress levels were found to be associated with increased experiences of minority stress, including discrimination in various aspects of life and in cancer care, discomfort related to one’s LGBTQI+ identity, lower disclosure of LGBTQI+ identity, and limited social support within these subgroups. These findings, based on the largest sample of LGBTQI+ individuals with cancer to date, highlight the diversity within LGBTQI+ populations in terms of health outcomes and provide valuable insights into the underlying mechanisms contributing to negative psychosocial outcomes for LGBTQI+ cancer survivors.

In a qualitative paper derived from the Out with Cancer Study, Power et al. examine the historical and contemporary experiences of discrimination, violence, family rejection, and exclusion that have created a legacy of distress and fear among LGBTQI+ individuals with cancer. The authors explore how these experiences have affected the level of trust towards healthcare professionals and contributed to distress and unmet needs in the context of cancer survivorship and care. Additionally, they investigate how social support from partners and chosen family members has mitigated the adverse impacts of minority stress, aiding LGBTQI+ individuals in coping with cancer. The study also highlights the agency and resistance demonstrated by LGBTQI+ patients and carers through collective action and advocacy. By shedding light on the unique socio-political histories and present-day psychosocial experiences of LGBTQI+ communities, this paper provides valuable insights into the factors contributing to distress during the cancer journey.

Understanding the intersectionality of identities is crucial for comprehending the experiences of LGBTQI individuals throughout their cancer journeys. Bates et al. draw upon the findings of the Restore-1 Study, and report sexual minority men of color, when compared to their white counterparts, experience lower health-related quality of life (HRQOL) scores in various domains, including bowel function, hormonal summary, hormonal function, and hormonal bother. This exploratory study provides initial evidence suggesting that sexual minority men of color may experience worse HRQOL outcomes following prostate cancer treatment compared to white, non-Hispanic sexual minority men. Rosser et al. present findings from the Restore-2 Study, which found gay or bisexual men (GBM), in comparison to heterosexual men, experienced significantly worse bowel, urinary, and hormonal function, and better sexual function and similar bother scores, aligning with previous research but in a larger sample. Additionally, GBM individuals had poorer mental health outcomes and worse quality of life. These findings highlight the presence of health disparities among sexual minority patients following prostate cancer treatment.

Research has consistently shown that GBM have higher rates of sexually transmitted infections (STIs) compared to heterosexual men throughout their lives. Moreover, evidence suggests that GBM may employ various strategies to manage sexual dysfunction, which can potentially increase the risk of acquiring STIs. Wheldon et al draw on the Restore-2 study and identify several risk factors for STI diagnosis, including engaging in non-monogamous sexual relationships, time elapsed since prostate cancer diagnosis, receiving penile injection treatment, reporting better sexual function, and having multiple sexual partners. These findings underscore the importance of integrating STI prevention into cancer survivorship plans, particularly as GBM regain sexual function over time.

The challenge of reaching LGBTQI+ populations affected by cancer is widely recognized. Myers et al. outline a multi-faceted, cost-effective, and systematic approach employed to engage LGBTQI+ communities in research, including methods to identify and filter out potentially fraudulent or suspicious online responses, ensuring data integrity. Among the strategies utilized, social media emerged as the most effective method for recruitment, surpassing direct mail outs. These study findings highlight successful strategies to effectively reach communities, enhance data quality, and mitigate the misrepresentation of data, which is crucial for improving health outcomes within LGBTQI+ communities.

During the challenging experience of being diagnosed with cancer, LGBTQ+ children and adolescents are also in a crucial stage of self-discovery regarding their gender identity and sexual orientation. Gannon et al explore the attitudes, knowledge, nd behaviors of pediatric, teenage, and young adult oncology HCPs treating LGBTQ+ patients in the UK. Using semi-structured interviews with eight HCPs, ten themes were revealed, including novel ones related to knowledge acquisition and reliance on a ‘third party’ as an expert. Specific concerns for LGBTQ+ patient care in pediatrics were identified, such as the influence of parental dynamics and age-related barriers to disclosure. The study highlights the interconnectedness of HCP knowledge, attitudes, and behaviors and proposes a suggested framework to improve HCP-patient interactions in LGBTQ+ cancer care than spans individual HCP education and organizational change. “ Cloyes et al emphasize the importance of understanding the access and engagement of support systems within the social networks of young adult (YA) and LGBTQIA+ survivors and care partners affected by cancer. They found that LGBTQIA+ participants had less dense and cohesive support networks, with a higher concentration of LGBTQIA+ members. They also received more appraisal support, particularly from relatives, compared to non-LGBTQIA+ participants. These results demonstrate how tailored and easily accessible assessment methods offer valuable insights into how real-world support systems operate, leading to the development of culturally sensitive interventions that address specific strengths and unmet needs. Such interventions are particularly crucial for young adult (YA) and LGBTQIA+ survivors and care partners, who often receive inadequate support from formal services and are underrepresented in cancer research related to caregiving and social support.

In their study, Waters et al. examine the intensified financial challenges faced by adolescent and young adult AYA cancer survivors who identify as LGBTQIA+. The findings reveal that LGBTQIA+ AYAs experienced significantly higher levels of financial burden and reported poorer mental health outcomes, including heightened levels of stress, anxiety, and depression compared to non-LGBTQIA+ AYAs. The increased costs of cancer treatment combined with the disruptions caused by the COVID-19 pandemic contributed to significant financial stress, further exacerbating existing mental health difficulties. These results underscore the substantial financial burden and psychological distress experienced by LGBTQIA+ AYA survivors, underscoring the importance of research to address their specific challenges and alleviate financial strain and adverse mental health outcomes.

Interactions with HCPs, and HCP beliefs and practices related to LGBTQI+ culturally safe care, were explored in a number of papers. Pratt-Chapman et al explore responses to a measurement tool, the QUIRKS-Patient and QUIRKS- Provider scales, among patient and providers in the United States. The Quirks scales measures constructs for patients in the domains of SGM environmental cues, patient experience, and attitudes. The scales for health care providers assesses the clinic readiness to meet SGM healthcare needs, environmental cues for affirming care, attitudes and knowledge. Using a snowball sample, results showed clinicians reported affirming clinic cues more often than patients. Clinicians were also more likely to report asking their patients about preferences and values for care than patients recalled being asked about these things. Patients reported greater understanding and comfort as to why they were asked to provide information regarding sex assigned and birth and gender identity at higher rates than providers assumed they would. Clinician’s knowledge was better for patients who identified as gay as opposed to other orientations and gender identities. Overall, their results support the need for expanded and improved provider training in the health care needs of SGM patients across the cancer care trajectory.

Ussher et al report draw on accounts of patient-HCP interactions from the perspective of LGBTQI+ patients, their caregivers and health care professionals in Australia. They identified three HCP mindsets regarding LGBTQI patients. “Inclusive and Reflective” practitioners noted the vulnerability of patients and the need for affirming care. Clinicians who approached their patients with this belief created safety and respect for patients, allowing them to freely disclose their SOGI data and report satisfaction with cancer care. Those clinicians who were characterized as “Egalitarian” reported ethical responsibility to treat all the patients the same and did not see relevance in the collection of SOGI data. As such, LGBTQI specific information was not likely to be provided and created anxiety and dissatisfaction among patients and their caregivers “Anti-inclusive” clinicians responses were those who reported hostility and prejudice for LGBTQI patients thus creating environments where patients felt distress, judged, and dissatisfied with their care. The authors conclude that a wide range of strategies are needed to improve LGBTQI cancer care including culturally competent training, redesign of environments and treating safe spaces for SOGI disclosure.

Kano et al, assessed quality of life using quantitative PROMIS measures and qualitative interviews among dyads of SGM (sexuality and gender minority) patients with cancer and their informal caregivers and heterosexual/cisgender (H/C) patients and their informal caregivers from the United States, to compare perceptions and experiences. The quantitative results showed greater anxiety, depression and social isolation among SGM patients than H/C patients. However, H/C patients more fatigue and pain but more social support. In qualitative interviews SGM patients and caregivers reported anti-SGM stigma and discrimination during their cancer care experience. SGM dyads had more medical mistrust than H/C dyads. Regarding communication, SGM patients with cancer did experience high satisfaction once trust was developed with their care team but wished for the opportunity to have more direct discussion regarding their SGM status. While some differences were observed, there were also several commonalities. Both SGM and H/C dyads noted appreciation for their health care teams. All patients and caregivers used social networks of friends and family. All caregivers felt remiss at the lack of information and support for their loved one’s treatment, side effects and ways to deliver support. The authors conclude that improvements are needed in clinical care teams cultural humility and ways to support caregivers.

Kamen et al report how researchers at two cancer centers in the United States worked with a group of LGBTQAI stakeholders with lived experience of cancer care to develop a community-academic partnership. Using the ADAPT-ITT model to guide their community needs assessment, the goal was to identify evidence-based interventions that could be adapted to meet the community needs. With a multi-phase approach, beginning with an assessment phase, the council members described their experiences and concerns. Cancer caregiving was noted as a priority for a future intervention. During the decision-making phase, a literature review was conducted for interventions that focused on cancer caregiving, identifying 13 potential interventions. Each intervention was evaluated by the council members using a rubric. The FOCUS intervention was then adapted for the LGBTQAI community. In the next phase, adaptation, the council identified the primary mechanisms to be modified. As the project is on-going, Kamen et al report on a process used to establish adapted intervention through community-academic partnerships for the LGBTQAI population during the cancer experience.

Tamargo et al report on a survey conducted among the US Eastern Cooperative Oncology Group (ECOG) and the American College of Radiology Imaging Network (ACRIN), examining clinicians’ experiences with SGM patients with cancer. Tamargo et al report on the qualitative analysis of the four open-ended items from the survey. Findings indicated clinicians had little experience with SGM patients, particularly transgender patients. Using correct pronouns was also reported as challenging among the 490 clinicians responding to the survey. A minority of clinicians reported SGM patients were more difficult to provide care for suspecting that prior negative experiences with the healthcare system were more likely to result in hostile patients with negative attitudes. However, the majority of respondents reported actual and potential positive experiences with SGM patients during the cancer care experience. The authors report need for clinicians desire and recognize the need for expanded training particularly for end-of-life care issues and opportunities to build trust across the SGM community

## 3 Conclusion

The papers featured in this special edition of Frontiers provide further evidence supporting the urgent call by The American Society of Clinical Oncology (1) to address and understand the health disparities faced by LGBTQI+ individuals with cancer. It is essential for cancer research to include questions about sexual orientation, gender identity, and intersex variation in order to identify unmet needs and shed light on the experiences of LGBTQI+ individuals in cancer and cancer care. By doing so, we can bring visibility to this potentially vulnerable population. Additionally, it is crucial to recognize the intersectionality of identities and how they influence the experiences of individuals with cancer, with a specific focus on underrepresented groups such as trans (27), intersex (28), AYA (29), and racially/ethnically diverse [Bates et al.] individuals. Engaging LGBTQI stakeholders through research co-design can help ensure that research methods and interpretations are culturally competent and culturally safe (30).

It is of utmost importance that we prioritize the development of affirmative and inclusive cancer care for LGBTQI+ people (17). This involves creating content that addresses the unique needs and experiences of the LGBTQI community as a whole, as well as content tailored to specific sub-groups such as trans, intersex, and AYA cancer patients (16, 31). A common thread throughout the publications in the field of health care interactions is the recognition of the necessity for expanded clinician training in cultural humility. It is crucial to establish opportunities for building trust through partnerships with SGM patients with cancer, their caregivers, and the healthcare institutions they rely on for care. By doing so, we can increase the likelihood of LGBTQI+ individuals with cancer and their caregivers having their needs acknowledged and met. This will result in affirmative and inclusive cancer care for LGBTQI+ communities, ultimately leading to improved health outcomes and higher levels of satisfaction with the care they receive.

## Author contributions

All authors listed have made a substantial, direct, and intellectual contribution to the work and approved it for publication.
